# Supplementary material for: Association between Geriatric Nutritional Risk Index and Depression after Ischemic Stroke
Source: Nutrients. 2022 Jun 29;14(13):2698. doi: 10.3390/nu14132698 (PMC9268883; doi:10.3390/nu14132698)
Supplement: Supplementary file 1 [file nutrients-14-02698-s001.zip › nutrients-1772021-supplementary.pdf]

Supplementary Material

1 Supplementary Tables

1.1 Table S1. Associations of malnutrition with risk of depression among patients with first-ever stroke

	OR (95%CI)	*P* value
Model 1	0.823 (0.472, 1.434)	0.492
Model 2	0.905 (0.502, 1.629)	0.738
Model 3	0.869 (0.455, 1.661)	0.671

Model 1: unadjusted model; Model 2: adjusted for age and sex; Model 3: adjusted for age, sex, education, hypertension, diabetes mellitus, heart disease, eGFR, admission NIHSS score, stroke subtype, and r-tPa treatment.

1.2 Table S2. Associations of GNRI scores with risk of depression among patients with first-ever stroke

	Tertile 1 (< 97.7)	Tertile 2 (97.7 – 101.4)	Tertile 3 (≥101.4)	*P* trend	Continuous (per SD increase)
Model 1	0.946 (0.493, 1.816)	0.789 (0.392, 1.159)	1.00	0.865	0.914 (0.688, 1.214)
Model 2	1.055 (0.524, 2.125)	0.807 (0.391, 1.677)	1.00	0.850	0.876 (0.634, 1.209)
Model 3	1.157 (0.524, 2.557)	0.761 (0.340, 1.704)	1.00	0.843	0.901 (0.629, 1.290)

Model 1: unadjusted model; Model 2: adjusted for age and sex; Model 3: adjusted for age, sex, education, hypertension, diabetes mellitus, heart disease, eGFR, admission NIHSS score, stroke subtype, and r-tPa treatment.

1.3 Table S3. Associations of different levels of nutritional risk with risk of depression among patients with first-ever stroke

Nutritional risk	Model 1		Model 2		Model 3	
	OR (95%CI)	*P* value	OR (95%CI)	*P* value	OR (95%CI)	*P* value
Without risk (n=139)	1.522 (0.673, 3.443)	0.313	1.492 (0.645, 3,451)	0.350	1.647 (0.654, 4.146)	0.290
Mile risk (n=53)	Reference		Reference		Reference	
Moderate risk (n=97)	1.351 (0.569, 3.207)	0.496	1.630 (0.654, 4.062)	0.294	1.857 (0.673, 5.129)	0.232
Severe risk (n=2)	Excluded		Excluded		Excluded	

Without risk: GNRI ≥ 100; mild risk: GNRI, 97.50–99.99; moderate risk: GNRI, 83.50–97.49; severe risk: GNRI < 83.50.

Patients with severe risk were excluded due to the small sample size.

Model 1: unadjusted model; Model 2: adjusted for age and sex; Model 3: adjusted for age, sex, education, hypertension, diabetes mellitus, heart disease, eGFR, admission NIHSS score, stroke subtype, and r-tPa treatment

1.4 Table S4. Associations of malnutrition with risk of depression among patients with mild stroke (NIHSS ≤ 4)

	OR (95%CI)	*P* value
Model 1	0.540 (0.285, 1.020)	0.058
Model 2	0.559 (0.284, 1.102)	0.093
Model 3	0.565 (0.273, 1.170)	0.124

Model 1: unadjusted model; Model 2: adjusted for age and sex; Model 3: adjusted for age, sex, education, hypertension, diabetes mellitus, heart disease, history of stroke, eGFR, admission NIHSS score, stroke subtype, and r-tPa treatment.

1.5 Table S5. Associations of GNRI scores with risk of depression among patients with mild stroke (NIHSS ≤ 4)

	Tertile 1<br>(< 97.1)	Tertile 2<br>(97.1 – 101.4)	Tertile 3<br>(≥101.4)	*P* trend	Continuous<br>(per SD increase)
Model 1	0.730 (0.347, 1.536)	0.659 (0.305, 1.426)	1.00	0.756	1.050 (0.771, 1.432)
Model 2	0.741 (0.329, 1.666)	0.621 (0.275, 1.404)	1.00	0.800	1.045 (0.741, 1.474)
Model 3	0.791 (0.320, 1.956)	0.562 (0.231, 1.368)	1.00	0.925	1.018 (0.702, 1.476)

Model 1: unadjusted model; Model 2: adjusted for age and sex; Model 3: adjusted for age, sex, education, hypertension, diabetes mellitus, heart disease, history of stroke, eGFR, admission NIHSS score, stroke subtype, and r-tPa treatment.

1.6 Table S6. Associations of different levels of nutritional risk with risk of depression among patients with mild stroke (NIHSS ≤ 4)

Nutritional risk	Model 1		Model 2		Model 3	
	OR (95%CI)	*P* value	OR (95%CI)	*P* value	OR (95%CI)	*P* value
Without risk (n=127)	3.033 (1.022, 9.175)	0.050	1.126 (0.586, 3.698)	0.055	3.613 (1.050, 12.434)	0.042
Mile risk (n=45)	Reference		Reference		Reference	
Moderate risk (n=90)	1.888 (0.584, 6.110)	0.288	0.716 (0.607, 1.372)	0.241	2.320 (0.658, 8.181)	0.191
Severe risk (n=3)	Excluded		Excluded		Excluded	

Without risk: GNRI ≥ 100; mild risk: GNRI, 97.50–99.99; moderate risk: GNRI, 83.50–97.49; severe risk: GNRI < 83.50.

Patients with severe risk were excluded due to the small sample size.

Model 1: unadjusted model; Model 2: adjusted for age and sex; Model 3: adjusted for age, sex, education, hypertension, diabetes mellitus, heart disease, history of stroke, admission NIHSS score, stroke subtype, eGFR, and r-tPa treatment

2 Supplementary Figures

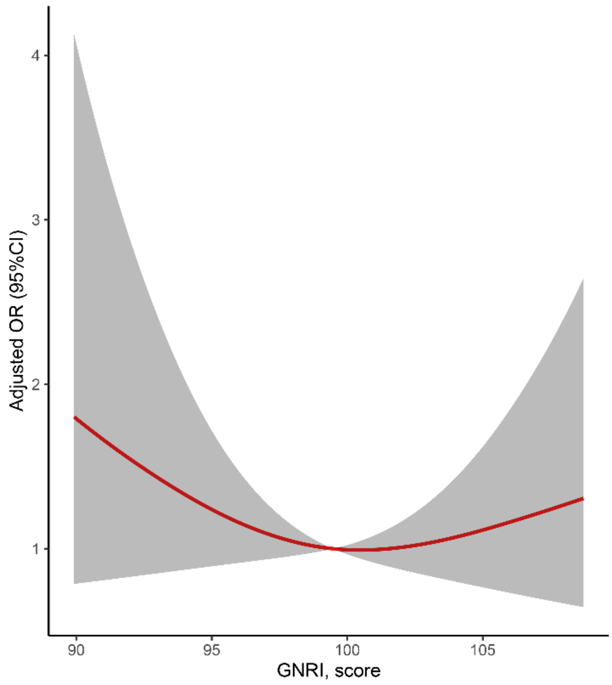

Figure S1. Relationship of GNRI and poststroke depression among patients with first-ever stroke. Odds ratios and 95% confidence intervals derived from restricted cubic spline regression (*p* for non-nonlinearity = 0.197), with knots placed at fifth, 50th, and 95th percentiles of the distribution of GNRI scores). The reference point was set at a GNRI value of 100. Odds ratios were adjusted for the covariates included in model 3 in Supplementary Table 1.

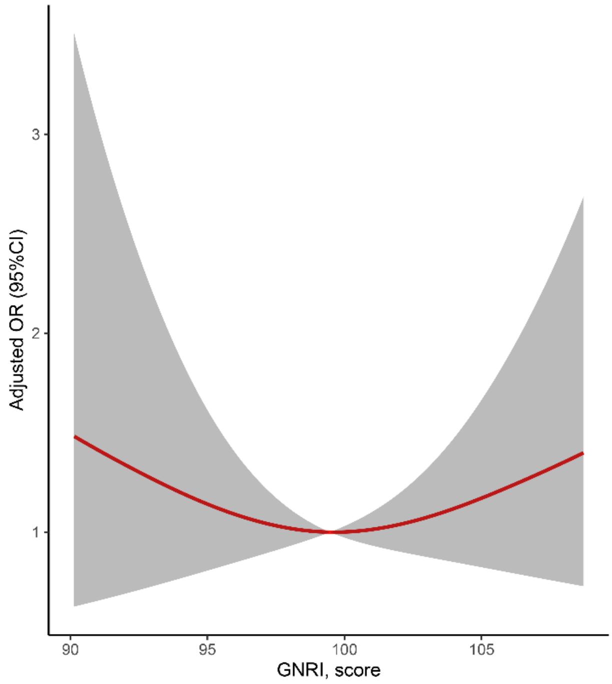

Figure S2. Relationship of GNRI and poststroke depression among patients with mild stroke (NIHSS ≤ 4). Odds ratios and 95% confidence intervals derived from restricted cubic spline regression (*p* for non-nonlinearity = 0.196), with knots placed at fifth, 50th, and 95th percentiles of the distribution of GNRI scores). The reference point was set at a GNRI value of 100. Odds ratios were adjusted for the covariates included in model 3 in Supplementary Table 4.
